# Delivery strategies to improve the pharmacological efficacy of NRF2 modulators: a review

**DOI:** 10.1039/d5md00571j

**Published:** 2025-08-22

**Authors:** Zerrin Sezgin Bayindir, Matej Sova, Nilufer Yuksel, Luciano Saso

**Affiliations:** a Faculty of Pharmacy, Department of Pharmaceutical Technology, Ankara University 06560 Ankara Turkey; b Faculty of Pharmacy, University of Ljubljana SI-1000 Ljubljana Slovenia; c Department of Physiology and Pharmacology “Vittorio Erspamer”, Sapienza University of Rome Rome Italy luciano.saso@uniroma1.it

## Abstract

The NRF2/KEAP1 signaling pathway regulates the gene expression of numerous cytoprotective and detoxifying enzymes and is therefore essential for maintaining cellular redox homeostasis. Despite the increasing knowledge of NRF2 signaling complexity, dimethyl fumarate remains the sole NRF2-targeting therapy in clinical practice, used for multiple sclerosis. Ongoing research exploring the role of NRF2 in cancer, neurodegeneration, diabetes, and cardiovascular, renal, and liver diseases holds significant promise for future therapeutic innovation. The therapeutic potential of NRF2 modulators, while supported by positive research and clinical data, is often restricted due to factors including low solubility, poor stability, poor pharmacokinetic parameters, and a lack of specificity that results in off-target effects. Therefore, designing an effective pharmaceutical formulation is one of the significant barriers to their clinical translation. This article addresses these challenges by reviewing various drug delivery strategies with a particular emphasis on polymeric nanoparticles, liposomes, polymeric micelles, carbon nanotubes, micro/nano-emulsions, and biomimetic nanoparticles. The potential of these systems to enhance the pharmacological activities of NRF2 modulators—driven by their small particle size and customizable properties—is discussed on a disease-by-disease basis, focusing on cancer, neurodegenerative, and inflammatory diseases. While these systems have shown considerable success in preclinical studies, their clinical application is constrained by hurdles in safety, scalability, stability and regulatory compliance. This transition has not yet been achieved for NRF2 modulators, but intensive research is ongoing. Therefore, the overall aim of this article is to provide a comprehensive understanding of delivery strategies for NRF2 modulators, ultimately guiding the development of more effective therapies and improving their clinical applications.

## Abstract 2

The NRF2/KEAP1 signaling pathway is a promising target for treating diseases. To overcome the poor formulation of NRF2 modulators, nanodrug delivery strategies are developed. While these systems show promise in preclinical studies, their translation into clinic requires further research.

## Graphical abstract

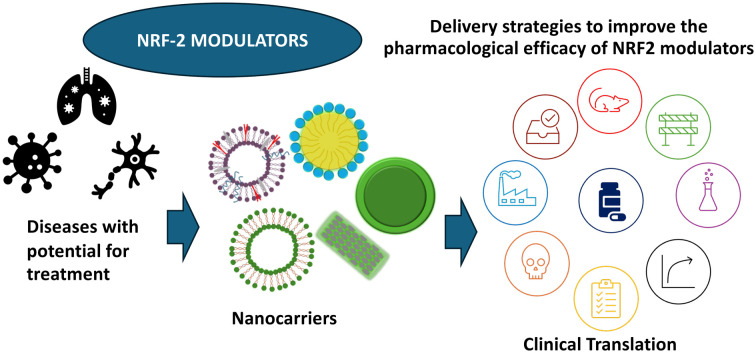

## 1. Introduction

The well-established role of Nuclear factor erythroid 2-related factor 2 (NRF2) in mitigating oxidative stress and enhancing cell defense underscores the therapeutic potential of NRF2 modulators. Their potential clinical benefit is enormous, especially where oxidative stress and inflammation are at the center of disease pathology. The modulation of NRF2 activity could therefore be beneficial in numerous diseases such as cancer, neurodegenerative diseases, diabetes, cardiovascular diseases and chronic respiratory, renal and liver diseases.^1^ Despite the broad clinical potential of NRF2 modulators, so far only dimethyl fumarate has been used in clinical practice for the treatment of moderate to severe plaque psoriasis and multiple sclerosis.^2^ Most recently NRF2 activator omaveloxolone has been approved for Friedreich ataxia.^3^ Many other NRF2 modulators such as sulforaphane, bardoxolone-methyl and oltipraz are currently being investigated in clinical trials,^2^ particularly for the treatment of cancer, diabetes and neurodegenerative diseases. On the other hand, there are some examples where NRF2 modulation has already failed in clinical translation. For example, the clinical trial on sulforaphane in chronic obstructive pulmonary disease showed that it did not stimulate the expression of NRF2 target genes when administered orally.^4^ Other formulations, *e.g.* inhalable formulations, may be more effective than oral administration. NRF2 also downregulates important inflammatory cytokines in COVID-19, but the clinical trial with sulforaphane did not show any improvement in hospitalized patients with suspected or confirmed COVID-19 infection.^5^ Another example is for bardoxolone methyl which did not reduce the risk of end-stage renal disease or death from cardiovascular causes in a clinical trial of patients with type 2 diabetes mellitus and stage 4 chronic kidney disease.^6^ In addition, formulation difficulties related to low solubility, poor bioavailability and stability also limit the clinical utility of NRF2 modulators. When formulated as nanomedicines, these compounds create innovative systems that reduce toxicity and boost the effectiveness of targeted therapies, offering significant clinical potential due to their high biological safety.^7^ This approach aligns with the growing interest in using nano drug delivery systems for NRF2 modulators to improve their clinical utility.

This review investigates the promising application of drug delivery systems to resolve the mentioned challenges, with a focus on recent nanomedicine developments for cancer, neurodegenerative, and inflammatory diseases. It concludes by discussing the clinical translation challenges associated with nanomaterials, such as premature content release, instability, nanotoxicity, and the need for standardized regulations.

## 2. Confronting oxidative stress

Electrophilic chemicals and reactive nitrogen/oxygen species (RXS) oxidize macromolecules and cell membrane lipids, potentially leading to abnormal function and cell death. Furthermore, they can also disrupt redox homeostasis and the regulation of redox signaling pathways. RXS act on organisms in a concentration-dependent manner, changing their activity from cytostatic to tumorigenic to cytotoxic as their concentration increases.^8,9^ This oxidative stress (OS) plays a role in numerous human diseases, including atherosclerosis, chronic obstructive pulmonary disease (COPD), idiopathic pulmonary fibrosis (IPF),^10^ Alzheimer-type diseases (ATD), cancer, and depressive disorders.^11^ Although several compounds tested as antioxidants have shown therapeutic promise in preclinical research, the results of clinical trials have been disappointing.^12^ A better understanding of the processes by which antioxidants function and the location and circumstances under which they are effective could provide a more rational strategy that translates into greater pharmacological efficacy.

Antioxidants inside cells play a key role in reducing OS and preventing cell death. NRF2 is one of the most effective cytoprotective controllers against OS. Impaired NRF2 activity is associated with numerous diseases, including cancer.^13^ It is a pleiotropic transcription factor^14^ that regulates the expression of genes involved in the antioxidant response, detoxification, wound healing,^15^ and cellular defense against OS, inflammation, and toxins. NRF2 belongs to the family of factors related to nuclear factor erythroid 2 p45.^16^

Forman and Zhang have extensively reviewed the relationship between OS, redox signaling and the ways in which OS can cause pathology, the mechanisms by which antioxidant defenses function, the factors that limit their efficacy, and the ways in which antioxidant defenses can be enhanced by physiological signaling, dietary elements, and potential pharmaceutical intervention.^12^ The authors draw several conclusions, which are outlined below. The mode of involvement of OS in different diseases is not the same, so that the increase in the effectiveness of defense mechanisms against OS may also vary with each pathology. Although clinical trials have not been encouraging, a thorough understanding of the mechanisms involved will undoubtedly improve outcomes. It is important to understand that the main defense of the organisms against OS is none other than enzymes as their action is essentially catalytic. Strategies include boosting antioxidant defenses and preventing the production of RXS by targeting their precursors, such as hydrogen peroxide (H_2_O_2_) and superoxide (O_2_˙^−^), although H_2_O_2_ has a critical function in physiological signaling. Attention must be paid to when OS plays a primary or secondary role in pathology to properly approach the investigation of each mechanism. Other aspects to bear in mind include the difficulty in achieving effective concentrations *in vivo*, the limited efficacy of non-enzymatic molecular scavengers, and the deteriorating ability to activate NRF2 with aging. The most effective strategy against OS is to prevent the production of the oxidants O_2_˙^−^, H_2_O_2_ and lipid hydroperoxides. Eliminating these species prevents the production of the more reactive hydroxyl radical (·OH), peroxynitrite (ONOO^−^) and hypohalogenated acids (HOX). The use of agents that remove H_2_O_2_ and O_2_˙^−^ from mitochondrial matrix and intracellular regions is a promising strategy. In the extracellular milieu, scavenging such precursors by superoxide dismutase (SOD), SOD-catalase, and glutathione (GSH) peroxidase (GPX) mimics have been shown to be helpful, and several of these drugs are now undergoing clinical studies. To maintain optimal levels of GSH, the natural substrate of GPX, precursors such as *N*-acetylcysteine (NAC) and GSH esters can be used. NAC is being used to treat various disorders in humans. Another strategy is to increase the production of natural antioxidant enzymes and *de novo* synthesis of GSH in cells through the activation of NRF2. The combination of all these methods will progress antioxidant treatments.^12^

**Fig. 1:**
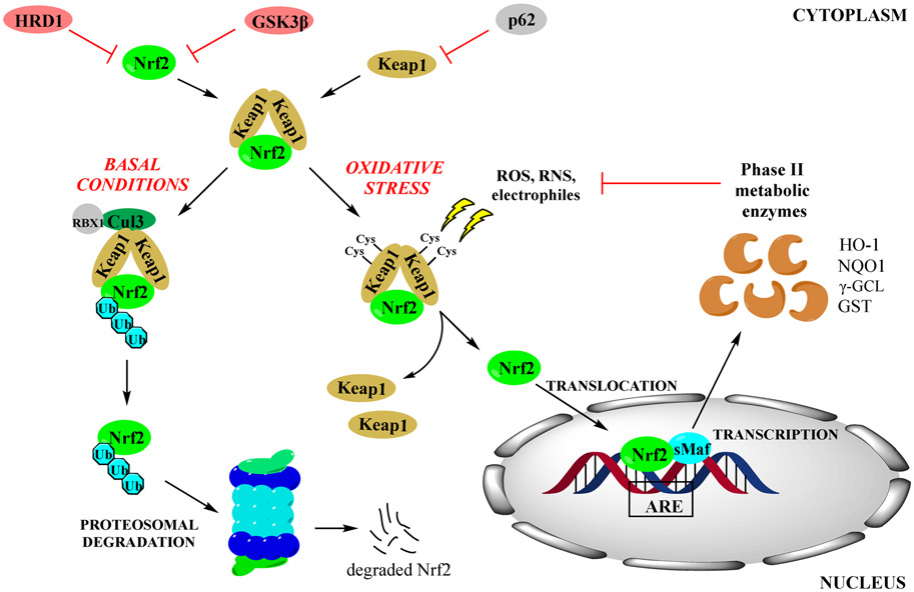
NRF2/KEAP1 signaling pathway in the basal state and under oxidative stress.

Cellular levels of NRF2 are extremely low in the absence of OS due to its continuous degradation (turnover: 20–30 min). This is maintained by three ubiquitination mechanisms induced by cytosolic Kelch-like ECH-associated protein 1 (KEAP1), by glycogen synthase kinase-3β (GSK3β) or by HMG-CoA reductase degradation 1 (HRD1) protein (Fig. 1). KEAP1 activates the cullin 3 scaffold protein, which brings the KEAP1-CUL3-RBX1 complex into play.^17^ The phosphorylation by GSK3β increases its affinity for β-TrCP, which causes the complex to be degraded by the SKP1-CUL1-RBX1 E3 ubiquitin ligase complex.^18^ NRF2 is degraded by HRD1 by interacting with its Neh4-5 domains.^19^ NRF2 concentration increases substantially in response to exposure to OS because electrophiles interfere with these mechanisms.^9,17^

KEAP1 binds strongly to NRF2 in the basal state, maintaining it inactive in the cytoplasm.^20,21^ KEAP1 acts similarly to IκB, which inhibits the activation and translocation of the transcription nuclear factor kappa B (NF-κB), which is involved in inflammation and immunity.^20,21^ KEAP1 is a redox-sensitive regulatory protein to which electrophilic agents, such as RXS, bind and form a Michael adduct *via* cysteine residues, in particular cysteine 151. This causes it to change its structure, which makes it unable to present NRF2 for degradation and release it (Fig. 1).^18^ On the other hand, OS inhibits GSK3β-mediated phosphorylation of NRF2, preventing their mutual interaction. Finally, the induction of autophagy degradation of KEAP1 by p62 also activates NRF2. These three events result in allowing NRF2 to enter into the nucleus.^12,20–22^ There, it interacts with members of the Maf or Jun family proteins and binds to DNA sequences known as antioxidant response elements (AREs) or, more properly, electrophilic response elements (EpRE), *cis*-acting elements (consensus sequence 5′-TGACnnnGC-3′) in the promoter regions of genes encoding “phase II” antioxidant enzymes.^20,21^ This triggers their transactivation. The phase II enzymes include hemeoxygenase-1 (HO-1), NAD(P)H quinone oxidoreductase 1 (NQO1), and γ-glutamyl cysteine ligase (γ-GCL).^23^ These enzymes effectively protect cells from damage, among other things by controlling the intracellular redox potential.^20,21^

The KEAP1/NRF2 signaling pathway has become the most important modulator mechanism of cellular homeostasis and a key player in cell survival and defense against oxidative and xenobiotic damage, *e.g.* in redox homeostasis, drug metabolism and excretion, detoxification, energy metabolism, iron metabolism, amino acid metabolism, inflammation, survival, proliferation, wound healing process, apoptosis, autophagy, proteasome degradation of oxidized proteins, DNA repair and mitochondrial physiology.^15,24–27^ NRF2 has been found to regulate the expression of more than 1000 protective genes, which constitute more than 1% of the human genome.^9^ Deregulation of KEAP1/NRF2 transcriptional activity has been linked to the pathophysiology of the numerous diseases.

For instance, NRF2 has been shown to reduce inflammation following lung injury.^28^ Its action is related to its ability to prevent nuclear factor kappa B (NF-κB) from migrating into the cell nucleus by any mechanism. NF-κB dependent gene expression is thereby inhibited. It has also been demonstrated that activation of NRF2 signaling pathway in alveolar macrophages can stop bacterial infections from exacerbating COPD. The function of NRF2 in pulmonary endothelial disorders like pulmonary hypertension is poorly understood. Nonetheless, it has been demonstrated that endothelial cells exposed to laminar shear stress induce NRF2. This pathway may be directly linked to the mitochondria and capable of sensing RXS generated from these organelles due to endothelial dysfunction.^28^

IPF is a chronic fibrotic condition characterized by an excessive fibroblast-to-myofibroblast transition induced by RXS and transforming growth factor-β (TGF-β), which presents extracellular matrix deposition. Macrophages are involved in fibrosis formation. NRF2 also regulates TGF-β expression. Thus, modulation of NRF2 signaling may be an effective treatment for fibrosis.^10^

Malignant cells can use this signaling pathway, which is the main drug detoxification pathway in normal cells, to evade the effects of numerous cancer treatments.^29^ Additionally, NRF2 also controls cell survival, differentiation, and growth.^30^

NRF2 modulators are being researched as potential treatments for disorders caused by OS, inflammation, and impaired antioxidant defense mechanisms.^31^ These include inflammatory disorders, cancer, and neurodegenerative diseases.^32^ For example, SARS-CoV-2 infection triggers a cytokine storm that causes severe tissue damage.^33^ Since the NRF2/NF-κB signaling pathways have been linked to the development of inflammation, NRF2 activator may be crucial for the treatment of COVID-19.^34^

As there are currently no inhibitors of the KEAP1/NRF2 pathway in clinical development, further preclinical research is needed to better understand the mechanisms of NRF2 modulation, in order to open the door to the development of safe and selective KEAP1/NRF2 modulators for novel therapeutic strategies.^9^

## 3. Therapeutic potential of NRF2 modulators and challenges

Targeting NRF2 signaling pathway holds great therapeutic potential since NRF2 is an important transcription factor that can influence and restore cellular redox and protein homeostasis, maintain mitochondrial function, and facilitate antioxidant, anti-inflammatory and detoxification responses.^35,36^ Therefore, NRF2 modulators could tackle various human diseases such as neurodegenerative disorders,^37^ autoimmune, metabolic and inflammatory diseases,^38^ cardiovascular and chronic respiratory, kidney and liver diseases,^39,40^ and cancer.^41^

Considering that NRF2 is an important regulator of OS defense, it provides protection and defense against various neurodegenerative conditions that occur in Alzheimer's disease, Parkinson's disease, multiple sclerosis, and amyotrophic lateral sclerosis. Because NRF2 is involved in the regulation of genes associated with antioxidant protection, autophagy, and proteasome activation, it represents an important target for NRF2 activators that could improve the treatment outcomes in neurodegenerative diseases.^37^ The next important feature of NRF2 is its ability to regulate immune cells in terms of differentiation, expansion, and survival as well as the cytokine release.^38^ Its immunomodulatory role is particularly important for the prevention of autoimmune diseases such as multiple sclerosis, systemic lupus erythematosus (SLE), and rheumatoid arthritis (RA). A series of studies in mouse models of SLE, summarized by Barati and Caster,^42^ have highlighted the therapeutic potential of NRF2 activators as they can attenuate oxidative and inflammatory stress and reduce tissue damage. Similarly, NRF2 activation resulted in anti-inflammatory and antioxidant effects in animal models of RA and in human RA synovial fibroblasts.^43^

Oxidative stress and inflammation are also common pathological features of many other chronic diseases. Since NRF2 is significantly involved in these processes, NRF2 modulators could attenuate ROS- and inflammation-induced stress in target organs such as the lung, kidney, and liver.^44^ It is also known that insufficient NRF2-dependent gene expression is implicated in various stages of cardiovascular disease. Indeed, many NRF2 activators have shown beneficial effects in various cell lines and animal models of cardiovascular disorders, such as attenuation of endothelial dysfunction, downregulation of inflammatory and pro-oxidant genes, reduction of systemic and vascular oxidative stress, suppression of vascular smooth muscle cell proliferation, prevention of cardiomyopathy, *etc.*^45^

Last but not least, NRF2 plays a critical role in cancer, acting as a double-edged sword.^29,46^ NRF2 can act as a proto-oncogene and as a tumor suppressor. Depending on the stage of the cancer, NRF2 activators or inhibitors can be used for treatment. Both NRF2 and by-products of OS, such as RXS and 4-hydroxynonenal (HNE), are involved in carcinogenesis.^47^ During conventional cancer treatments, such as radiotherapy and chemotherapy, cancer cells experience OS, generating RXS and HNE, which contribute to cell death. Although NRF2 was initially considered as a tumor suppressor that inhibits cancer development and spread, substantial evidence reveals its pro-oncogenic role. The increased expression of NRF2 in various types of cancer supports its involvement in unfavorable pathophysiological functions. Cytoprotective genes are activated as a result of NRF2 overexpression in cancer cells, which enables malignant cells to withstand high levels of RXS and evade apoptosis, ultimately making them resistant to standard cancer therapies. Therefore, alternative therapeutic approaches are needed to effectively eradicate cancer.

Nevertheless, several activators of the NRF2 antioxidant response element have been tested as chemopreventive therapies for OS-related disorders, including cancer. According to research, hyperactivation of the NRF2 signaling pathway leads to a scenario that aids the survival of both normal and cancer cells, protecting them from OS, anticancer drugs and radiation. Telkoparan-Akillilar *et al.* have addressed the regulation of the NRF2 signaling pathway, anticancer activity, and the difficulties in developing NRF2-based cancer therapeutic options.^16^ Novel technologies such as CRISPR/Cas9 was used to disable the NRF2 gene in chemoresistant lung cancer cell.^48^ The combined action of CRISPR-directed gene editing and chemotherapy increased effectiveness of the anticancer drugs cisplatin, carboplatin, and vinorelbine. CRISPR/Cas9-based modulation of NRF2 could thus be used in sensitization of cancer cells to chemotherapeutic drugs.

Sova and Saso have reviewed in depth several design approaches used to develop NRF2 modulators.^13^ They have also focused on the most important and recently developed NRF2 activators and inhibitors, their *in vitro* and *in vivo* research, and their potential utility as cancer therapies or chemopreventive drugs. Molecules like dimethyl fumarate (DMF), sulforaphane, and oltipraz, natural substances like curcumin, and synthetic compounds like GSK-3 inhibitors and proteasome inhibitors have all demonstrated potential for controlling NRF2 activity.^49^ For instance, dihydroartemisinin has been shown to potentially enhance the efficacy of cytarabine, a standard chemotherapy drug, in acute myeloid leukemia cells. This effect was attributed to the suppression of NRF2 transcriptional activity.^50^

DMF has been shown to act on the cellular inflammatory response pathway^51^ and has been used in the treatment of psoriasis and multiple sclerosis. The European Community has approved the formulation Fumaderm® for topical application for dermatological problems.^28^ Another formulation, in the form of a delayed-release oral capsule called Tecfidera® has been approved in the USA for multiple sclerosis.^28,52^ DMF is currently being investigated for use in other inflammatory and autoimmune diseases, such as polyarthritis,^28^ vascular calcification,^53^ renal fibrosis,^54^ pancreatitis^55^ and COPD.^56^

Polyphenols, such as quercetin and epigallocatechin-3-gallate, and quinones, such as *p*-terphenylquinone and seriniquinone, have also been recognized as modulators of OS. A review by Kostenko and co-authors compiled the effects of polyphenols on specific transcription factors and highlighted their potential in controlling low- and high-grade systemic inflammatory responses, emphasizing the need for targeted delivery systems and further research on these treatments.^57^ A study by Lin *et al.* has shown that polyphenols inhibit the doxorubicin cardiotoxicity in rodents caused by increased RXS formation, enhanced inflammatory response and mitochondria-mediated apoptosis, and suppression of the NRF2 signaling pathway.^58^ Similar conclusions have been reached by Wang and co-authors using cardamonin as a protective agent.^59^ As for quinones, Nagao and co-authors have synthesized a series of *p*-terphenylquinone and seriniquinone derivatives and tested them on human leukemia HL-60 cells. Some of the tested quinones showed higher antiproliferative activity than seriniquinone so authors emphasize the potential of this type of derivatives as anticancer agents that modulate ROS induced by redox cycling.^60^ The Traditional Chinese Medicine of Dantongding exhibits therapeutic potential by providing anti-inflammatory and analgesic effects in ameliorating hyperglycemia-induced diabetic peripheral neuropathy (DPN) in rats. It exerted this effect by activating the Nrf2/HO-1 pathway and inhibiting the NF-κB pathway.^61^

Wang *et al.* used an N2a/APP cell model of ATD to investigate in detail the neuroprotective potential of the PcActx peptide from zoantharian *Palythoa caribaeorum* and its underlying mechanism of action, an antagonist of the transient receptor potential cation channel subfamily V member 1 (TRPV1).^62^ The PcActx peptide significantly reduced the expression of BACE1, PSEN1 and PSEN2 and the formation of amyloid-related proteins, as shown by Western blot and RT-PCR analyses. In addition, the PcActx peptide blocked CaMKII and CaMKIV (calcium-mediated proteins) phosphorylation in N2a/APP cells, as well as the capsaicin-induced calcium response. Further studies revealed that PcActx peptide greatly reduced RXS production by activating NRF2, which led to an increase in NQO1 and HO-1 levels. Furthermore, PcActx peptide drastically reduced PcActx-induced NRF2 activation and amyloid dysregulation, while it significantly increased Akt phosphorylation at Ser 473 (active) and Gsk3 phosphorylation at Ser 9 (inactive). In conclusion, the PcActx peptide functions as a TRPV1 modulator of intercellular calcium homeostasis, inhibits ATD-like amyloid neuropathology through Akt/Gsk3-mediated NRF2 activation, and represents a potential alternative agent for the treatment of ATD.^62^

Ershov and his co-authors have reviewed the current state-of-the-art in the field of interfacial peptides as potent modulators of a number of disease-related protein–protein interactions, including NRF2/KEAP1.^63^ The creation of modified peptide structures has triggered a new wave of research. Selected findings from this review are summarized below. Lu *et al.* have rationally designed and synthesized a KEAP1-ligand cyclic interfacial peptide that uses Gly as a linker for the C- and N-terminal ends.^64^ To design the ligand, they started from a nonapeptide with known high affinity for KEAP1, studied its detailed binding mode, attempted to improve its properties and adding residues to make the cyclization feasible, guided by molecular dynamics. Lu *et al.* claimed that the peptide enhanced NRF2 activation at the cellular level.^64^ However, re-examination by other authors revealed that this cyclo-peptide has “weak cell-penetrating activity”.^65^ According to another study, perfluoroarene-based peptide macrocycles were able to block the interaction between NRF2 and KEAP1 because the disulfide and perfluoroalkyl bridges in the structure of the peptide mimicking the beta-turn of KEAP1 restricted conformational flexibility.^66^

Despite of high therapeutic potential of NRF2 modulators, there are still many challenges that need to be addressed/overcome before their clinical use. The main obstacles to enhance practical applicability of NRF2 modulators are their target specificity, toxicity, low bioavailability, instability, and poor solubility.

NRF2 activators can act *via* two different mechanisms: i) binding to the Cis KEAP1 residues and triggering NRF2 release and ii) blocking the KEAP1 interface. The former can be termed as indirect activators, while the latter would be direct activator.^67^ Direct NRF2 activators are NRF2/KEAP1 protein–protein interaction inhibitors, while indirect NRF2 activators are electrophilic agents that induce a conformational change of KEAP1 after interaction with the cysteine residues, thereby impeding its binding to NRF2 and consequently reducing NRF2 degradation.^68^ The problem with indirect activators lies in their ability to react with other proteins that have exposed cysteine, which makes them less selective.^39^ Therefore, they can affect the function of enzymes and proteins, leading to “off-target” and toxic effects. For example, the indirect activator DMF showed adverse gastrointestinal effects that may lead to discontinuation of treatment.^69^ Direct inhibition of Keap1–NRF2 protein–protein interactions has therefore become an attractive strategy for the activation of NRF2. Other alternative approaches such as adeno-associated viral-mediated delivery, antisense oligonucleotides, and improved delivery systems are also considered as viable options.^70^ Another example was presented by Muralidharan *et al.*, who proposed to use inhaled delivery to target indirect NRF2 activators to the desired site of action in the lungs, thereby reducing or eliminating off-target adverse effects.^71^

Many NRF2 modulators also suffer from poor pharmacokinetic properties, particularly in terms of low bioavailability, instability, and poor water solubility. One of the most studied classes of natural NRF2 modulators is phenolic compounds.^72^ The majority of polyphenols have shown low bioavailability due to their poor water solubility. For example, the natural NRF2 activator curcumin has low bioavailability due to its poor solubility in water, low permeability and absorption, and rapid metabolism.^73^ The situation is similar with another natural NRF2 activator resveratrol, which is also poorly soluble in water and also exhibits instability, auto-oxidation and rapid metabolism. Another example of NRF2 activators are anthocyanidins, which are insoluble at the intestinal pH. However, these compounds can be modified by the intestinal microbiota into easily absorbed metabolites that still possess antioxidant activity. One might think that polyphenols could be easily supplied by food, but they are easily destroyed during cooking, which makes their effective concentration in cooked foods very low.^74^

Grilc *et al.* have presented an overview of the most common formulations to improve the oral bioavailability and *in vivo* pharmacokinetics of NRF2 modulators.^75^ They also briefly present the cellular and molecular mechanisms involved in NRF2 modulation. Solid dispersions, self-microemulsifying drug delivery systems and nanotechnological methods, such as solid and polymeric lipid nanoparticles, nanocrystals and micelles, are some of the formulations that have also been examined *in vivo*. Finally, nanodevices for the delivery of NRF2 modulators to the brain are briefly discussed. The various formulations can improve the pharmacokinetics and bioavailability of natural NRF2 modulators. This has been demonstrated in both animal models and human research, increasing the likelihood of natural NRF2 modulators being used clinically.

With regard to peptide inhibitors of KEAP1, Colarusso *et al.* have developed a library of linear and cyclic interfacial peptides, against NRF2/KEAP1 that have shown high binding capacity to KEAP1 and the ability to release NRF2, but they were not active in living cells due to lack of cell permeability.^76^ In addition to selective binding to cellular targets, the development of peptide modulators often poses particular problems in terms of targeted delivery, cell penetration and proteolytic stability.

Another critical obstacle that has also hindered the application of several promising NRF2 activators for neurological pathologies is the ability of the drug to cross the blood–brain barrier. Potential approaches to overcome this obstacle include mild disruption of the integrity of the blood–brain barrier, modification/tagging of established agents to improve their stability and penetrance, and intranasal administration to completely bypass the blood–brain barrier.^70^

In addition to target specificity, undesired side effects and poor pharmacokinetic properties, there are also other challenges in targeting NRF2, which were summarized in a review by Dinkova-Kostova and Copple.^35^ The development of NRF2 modulators for therapeutic use would require the appropriate disease selection and adequate monitoring of pharmacodynamic responses in patients. The route and frequency of administration, dose and appropriate delivery systems will also be a major challenge in the future development of NRF2 modulators.

## 4. Drug delivery strategies for NRF2 modulators

The goal of any drug delivery system is to transport and maintain therapeutic drug concentrations to specific tissues, organs, cells, and subcellular organs for drug release and absorption. They aim to increase the pharmacological efficacy and solve problems such as limited solubility, drug aggregation, low bioavailability, poor biodistribution, lack of selectivity, and adverse effects.^77^ Drug delivery techniques have contributed significantly to the transformation of potential drugs into effective treatments. Delivery methods and technologies have adapted to evolving drug delivery requirements as the therapeutic landscape has changed. A few decades ago, small molecule drugs were the main therapeutic category.^78^ Since the bioavailability of small molecules is highly dependent on their physicochemical properties, delivery efforts initially focused on increasing the solubility of drugs, managing their release, prolonging their activity, and modifying their pharmacokinetics. Poor aqueous solubility causes low drug dissolution rates in body fluids, which in turn leads to low drug concentration in the blood. Because of their insoluble nature, NRF2 modulators have poor *in vitro*–*in vivo* correlation, which often contributes to their termination during the clinical stage. Therefore, the use of conventional solubilization techniques (such as solubilizers, cosolvents, prodrugs, and micronization) and new strategies (like advanced drug delivery systems) is often necessary for solubility enhancement.^79^ The *in vitro* and *in vivo* stability of NRF2 modulators highly depends on their structure. In this respect, it is known that modulators with polyphenol structures show significant differences.^80^ Instability against temperature and light leads to low physical and storage stability, which is also influenced by pH and oxygen. The harsh gastric medium and enzymatic degradation contributes to their low *in vivo* stability. For example, in an *in vitro* digestion model, the polyphenol epigallocatechin was shown to remain at a low level of only 4.6% after passing through salivary and gastric fluids and reaching the upper small intestine.^81^ Although the plasma half-life of NRF2 modulators is variable, this period is often short for many known modulators (*e.g.*, 3.5 hours for quercetin).^82^ This makes the dosing regimen adjustment and patient adherence challenging and make it imperative to develop effective formulation strategies.

**Fig. 2:**
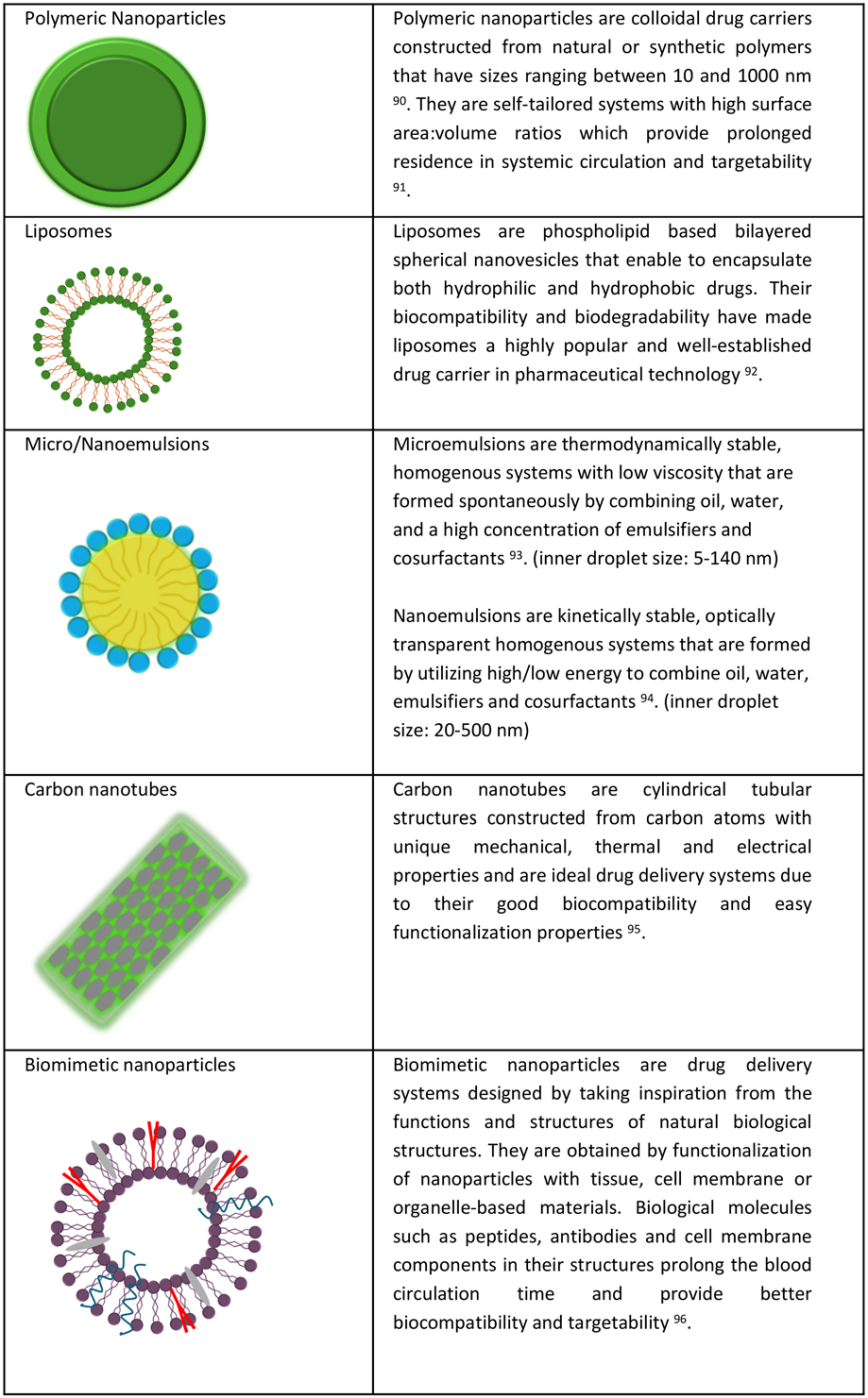
Illustration and description of nano-drug delivery systems developed for the use of NRF2 modulators in various medical treatments.^90–96^

Nanoparticles have become one of the most promising drug delivery technologies in recent years.^83,84^ The therapeutic index increases, and the probability of side effects decreases when drugs are loaded in nanoparticles, such as micelles, liposomes, dendrimers, nanocapsules, and nanospheres. Fig. 2 illustrates different nanosystems studied for biomedical purposes. As the drugs are loaded in nanoparticles, their physical and biological stability, and solubility are improved. The large surface area of these systems due to their small particle size provides unique mechanical, magnetic, optical, and catalytic properties, which make them promising drug carriers.^85^ Due to their biocompatible and biodegradable properties, most of the nanoparticles (such as liposomes, micelles *etc.*) can be safely metabolized and eliminated over time. The surface of nanoparticles can be engineered with different moieties (such as polymers, antibodies, folate, glucose, *etc.*) to provide cell targeted drug delivery, and improved cell–drug interactions. They can be easily absorbed through biological membranes, attributing to their nanosize and when combined with their capacity for targeted delivery they provide significant therapeutic advantages. The drug loading capacity and release kinetics of the loaded therapeutics can be controlled by modulating the composition, size, and surface charge of the nanosystems.^86^ Besides, emerging strategies for drug delivery include stimulated release. This approach, which can benefit from endogenous or exogenous activators, has been actively investigated for tumor-specific delivery and controlled release of their payloads. Endogenous activators include those that are sensitive to temperature, enzyme, reactive oxygen species, pH and redox potential. Exogenous activators include temperature, light, X-rays, magnetic fields and ultrasound.^87^ Focused ultrasound has the advantage of generating spatially confined heat, which favors site-specific controlled release by rendering the structures of these release systems unstable.^88^ Endosomal degradation poses a significant challenge to nanoparticle stability after cellular uptake; however, the engineering nanoparticulate systems (*e.g.* pH sensitive systems) allows for endosomal escape to deliver the cargo to the cell cytoplasm, which ultimately increases the success of drug and gene therapies. However, plain nanoparticles face several challenges, including uptake by the reticuloendothelial system, pro-oxidant formation, and insufficient targetability. Consequently, recent research has focused on developing biomimetic drug delivery systems. These systems are designed to overcome biological barriers by utilizing the body's own natural compounds. They are created by functionalizing nanoparticles with materials derived from tissues, cell membranes, or organelles, and they contain biological molecules such as peptides, antibodies, and membrane components. This biomimetic approach gives synthetic nanoparticles biological characteristics, allowing them to evade the immune system, circulate longer in the bloodstream, exhibit higher biocompatibility, and achieve greater targeting success. In light of these advantages and the available literature, it is seen that formulation of NRF2 modulators using nano drug delivery systems has the potential of improving their clinical usage.

The analyses in the present article include examples of recent NRF2-based research conducted specifically for various disease profiles.

### 4.1. Drug delivery strategies in cancer treatment

Cancer is an important area of scientific research due to its high prevalence and mortality. Regulating NRF2 activity through inhibition or activation offers a promising approach to cancer prevention and personalized therapy, reflecting its dual role as both a tumor suppressor and a pro-oncogenic factor. In particular NRF2 inhibition can trigger reactive oxygen species (ROS)-induced cell death in various cancers. However, the previously mentioned challenges such as low solubility limit their potential usage. We have previously conducted a comprehensive review of nanoparticles' role in augmenting NRF2 modulators efficacy in cancer treatment.^89^ Solubility enhancement *via* encapsulation of NRF2 modulators in nano drug carriers such as liposomes, nanoemulsions was shown to improve *in vivo* bioavailability. Enhanced permeability and retention (EPR) effect is the main phenomenon that explains the accumulation of nanoparticles in tumors due to the leaky nature of tumor blood vessels and impaired lymphatic drainage. The contribution of the small size of the nanoparticles is the key factor in the EPR effect. Improved cell cytotoxicity cell culture models and tumor growth inhibition in animal models were shown for NRF2 loaded nanosystems. Some of the recent studies on related topic are given in Table 1.

**Table 1:** Recent research investigating the use of NRF2 modulator-loaded drug delivery systems for cancer treatment

NRF2 modulator/drug	DDS	*In vitro*/*in vivo* results	Ref.
Quinacrine	Bovine serum albumin (BSA) decorated lipidic core nanoparticles	In the study, quinacrine-loaded nanoparticles inhibited NRF2 and provided an increased synergistic interaction with low-dose docetaxel which led to cell apoptosis. Thus, the anti-proliferation activity of docetaxel against A549 lung cancer was improved	97
Melatonin	MIL-68(Al) nanoparticles (metal–organic frameworks-MOFs)	Sustained melatonin release was obtained *via* drug loading into nanoparticles. The cytotoxicity and anti-proliferative effects of melatonin in A549 lung carcinoma cells was significantly improved compared to free drug and cytotoxicity was low in normal cells. Overall results were attributed to effective suppression of the NRF2/Keap-1 pathway	98
Cisplatin and anthocyanin	Multiwalled carbon nanotubes coated with chitosan-conjugated folic acid	The combination therapy evaluated in MCF7 (breast) and HepG2 (liver) cancer cell lines revealed anticancer effects in terms of increased antioxidant status, higher cytotoxic and apoptotic effects. Besides, reduced TNF values revealed the attenuation of inflammation	99
Brusatol and docetaxel	Poly(lactide-*co*-glycolide) (PLGA) nanoparticles	Prostate specific membrane antigen (PSMA) – specific ligand was used to actively target the nanoparticles to cancer cells. Increased ROS generation in prostate cancer cells in cell culture and tumor growth inhibition was shown in athymic BALB/c mice implanted with PSMA-producing LNCaP cell tumors	100
Anti-EGFR antibody, brusatol, and NRF2-siRNA	Fe_3_O_4_@ZnO nanoparticle	UV-triggered drug release from nanoparticles provided chemo-, gene, and photodynamic therapy as a combination. The ROS levels were increased in cancer cells, and cell viability was reduced. Furthermore, external magnetic field application and anti-EGFR antibody provided active drug targeting in the carcinoma-bearing Balb/c nude mice and led efficient tumor reduction rate	101

### 4.2. Drug delivery strategies in the treatment of neurodegenerative diseases

Currently, with increasing life expectancy, the incidence of neurodegenerative diseases such as Alzheimer's, Parkinson's, multiple sclerosis, and similar diseases is also increasing. Neuroinflammation, mitochondrial dysfunction and oxidative stress are among the key features of neurodegenerative diseases.^37^ Therefore, as the regulator of antioxidant and detoxification genes Keap1/NRF2/ARE signaling pathway has a major role in treating neurodegenerative pathologies.^102,103^ As the first clinical reflection of this, an NRF2 activator, DMF, has been successfully used in multiple sclerosis treatment, which is characterized by demyelination and neuronal loss. Although the exact mechanism of action of DMF is unknown, it reduces the severity of disease symptoms *via* neuroprotection, immunomodulatory, and antioxidant effects. There are also studies on different NRF2 modulators, but no other application has yet reached the clinic, especially due to formulation difficulties.

In general, the inadequacy of current therapies, difficulties in overcoming biological barriers such as the blood–brain barrier (BBB), low solubility and stability of active substances, and low patient compliance, especially in invasive treatments, require more effective treatments and innovative solutions in neurodegenerative diseases.^104^ The invasive methods for treating neurological conditions may require the usage of alternative drug administration routes, including intracerebral, intrathecal, and intraventricular ways, which carry a high risk of infection. Implantable systems have the drawback of catheter-related complications. BBB disruption *via* ultrasound or electromagnetic radiation application or coadministration of hyperosmotic solutions are other possible approaches that have trauma risk. Therefore, non-invasive methods, including intranasal drug administration, using derivates of the active molecules with better bioavailability and advanced drug delivery systems, seem to be more feasible therapeutic strategies.^105^ The nano-engineered smart drug delivery systems lead brain-targeted drug delivery as they can pass through BBB, reach the central nervous system, and efficiently treat neurodegenerative diseases. The potential of nano drug delivery systems for the clinical translation of NRF2 modulators to treat neurodegenerative diseases is thoroughly studied (Table 2).

**Table 2:** Recent research investigating the use of NRF2 modulator-loaded drug delivery systems for neurodegenerative diseases

NRF2 modulator/drug	DDS	*In vitro*/*in vivo* results	Ref.
Dimethyl fumarat	Platelet-based nanoparticle	The limited brain penetration of the drug was improved *via* biomimetic nanoparticles. The pharmacokinetics and neuropharmacokinetic studies conducted on rats revealed improved brain uptake clearance	106
Pterostilbene	Nanoemulsion	The formulation reduced the oxidative stress within the hippocampus of rats with Aβ1-42-induced disease model. Consequently, hippocampal neuron injury was reduced, and improvement in learning and memory function was observed in the behavioral tests	107
Lithospermic acid B	Phosphatidylcholine liposome	Lithospermic acid B-loaded mannose-modified liposomes demonstrated enhanced BBB penetration and neuron targeting in HT22 and bEnd-3 cell models. The formulation provided significant protection against neurodegeneration, restored mitochondrial health, and alleviated memory deficits in transgenic mice with Alzheimer's disease	108
Brain-derived neurotrophic factor (BDNF)	Mesenchymal stem cell-derived exosomes	*In vitro* assessment of therapeutic efficacy of BDNF loaded exosomes in Parkinson's disease (PD) models showed the inhibition of both apoptosis and ferroptosis. *In vivo* application of exosomes *via* the tail vein of mice resulted in NRF2 activation, significant reduction of 6-OHDA-induced lipid peroxidation, and protection of dopaminergic neurons from ferroptosis. Overall results exhibit significant neuroprotective effects and potential of treating PD	109

### 4.3. Drug delivery strategies in in the treatment of inflammatory diseases

Inflammation has a key role in many chronic diseases and is critical in carcinogenesis. It is recognized that NRF2 exerts significant control over inflammation by regulating the recruitment and gene expression of inflammatory cells through the antioxidant response element (ARE). Plain or modified nanoparticles can be formulated for inflammation management *via* drug targeting.^110^ They can be designed for passive targeting *via* leaky vasculature or active targeting of key inflammatory components (such as macrophages, endothelial cells, membrane receptors, anti-inflammatory genes, and cytokines). The former can be achieved through nanoparticle functionalization with targeting moieties such as coating groups, antibodies, or affinity ligands (such as spermidine^110^). Beyond drug delivery, nanoparticles serve as vaccine adjuvants and modulate the immune system. Their shape is a critical factor that affect their interactions with the components of the innate immune system.^111^ Due to the above-mentioned difficulties of clinical usage of NRF2 modulators, scientific literature extensively documents the potential of nano drug delivery systems to facilitate their *in vivo* potential to treat inflammatory diseases (Table 3).

**Table 3:** Recent research investigating the use of NRF2 modulator-loaded drug delivery systems for inflammatory diseases

NRF2 modulator/drug	DDS	*In vitro*/*in vivo* results	Ref.
Procyanidin	Metal–phenolic networks (MPNs) loaded onto the surface AuAg nanoparticles (NPs)	It was shown that the drug delivery system with photothermal effect eliminates bacterial biofilm and plays an immunoregulatory role. *In vivo* studies in mouse models have shown the good therapeutic effect on periodontitis and significant amount of collagen fiber formation	112
Luteolin and dexamethasone	l-α-Phosphatidyl choline/hyaluronic acid based hyalurosomes	Dual drug carrying hyaluronic acid based nanoparticles were found out to be more effective on reducing the joint swelling and inflammation in the rat model of rheumatoid arthritis compared to positive control group. Therefore, an improved synergetic effect was obtained *via* the drug delivery system	113
Rhein	Drug-polyethylene glycol (PEG)–triphenyl phosphonium (TPP) congugate based micelles	Rhein was conjugated with polyethylene glycol (PEG) and triphenyl phosphonium (TPP) which self-assembled into a micellar drug carrier. The mitochondria-targeted rhein-loaded micelles were evaluated for the treatment of osteoarthritis. *Via* the NRF2 activator role of rhein, improved anti-inflammatory and antioxidative effects were obtained and the level of reactive oxygen or nitrogen species were decreased in cell culture studies	114
Dimethyl fumarat	Liposomes	Lui *et al.* have investigated liposomes for the administration of DMF by inhalation and the activation of macrophages liposomes have reduced both macrophage activity and fibrosis in mice. The co-culture of RAW264.7 and NIH-3T3 cells has shown that formulations could increase the expression of NRF2 and heme oxygenase-1 (HO-1) and suppress the generation of TGF and RXS in macrophages, which in turn reduces collagen formation by NIH-3T3 cells. Liposomes were more effective against pulmonary fibrosis than direct DMF, due to their longer lung residence, biocompatibility, and ability to reduce fibrosis *via* enhanced NRF2 signaling	10
Magnolol	Nanostructured lipid carriers	Magnolol, an NRF2 activator with established anti-inflammatory and antioxidant effects, faces challenges with oral administration due to poor solubility and bioavailability. Jia *et al.* addressed this by developing a nebulized nanostructured lipid carrier formulation, which successfully prolonged lung residence and significantly regulated inflammatory and oxidative stress markers in COPD models	115
Astaxanthin	Polylactic acid–glycolic acid nanoparticles	The nano-platform inhibited the macrophage ferroptosis by activating NRF2 pathway and enhancing oxidative stress. Therefore, the atherosclerosis was inhibited	116
Berberine	ZnO nanocolloids	Berberine-loaded ZnO nanocolloids have improved the rate of wound healing by increasing the expression of antioxidant factors such as Nrf2, HO-1 and NQO1, as well as reducing the production of proinflammatory cytokines such as TNF-α, IL-1 and IL-6 and increasing the level of vascularization-related factors such as VEGF and CD31	117

## 5. Challenges associated with clinical translation of nano drug delivery systems

Nanoparticle drug delivery and imaging systems are the most researched systems in preclinical and clinical terms. Depending on the desired application and target site, nanoparticles can be administered orally, topically, locally, and parenterally. Formulation of NRF2 modulators using nano drug delivery systems (such as bovine serum albumin decorated lipidic core nanoparticles, metal–organic nanoparticles, carbon nanoparticles, polymeric nanoparticles, *etc.*) has the potential of improving their clinical usage (Tables 1–3). There are many studies on nanomedicines and a bibliographic analysis of the WoS Core Collection showed that the number of nanomedical studies, which was approximately 2000 in 2000, increased by more than 10-fold to over 18 000 by 2019. One of the most significant research areas in nanomedicines over the past 20 years is drug delivery, accounting for 49% of publications and 76% of all nanomedical research and drug/therapies.^118^ Although the nanomedicine area continues to expand through new drug delivery strategies, new technologies, new treatment approaches, and poor clinical outcomes of existing drug molecules, the clinical translation and the number of formal approvals of nanomedicines have not increased to the same extent.

**Fig. 3:**
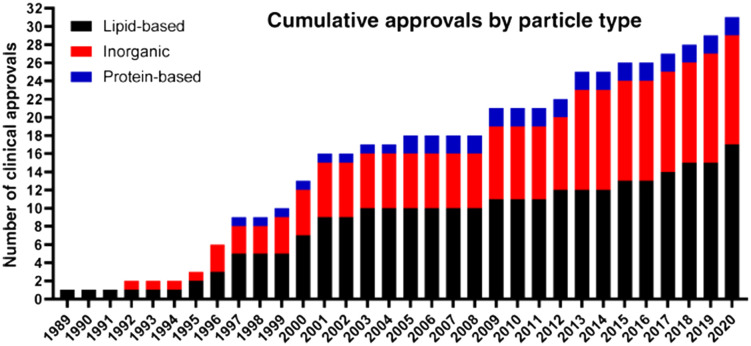
Distribution of approved particle-based formulations over the years (1989–2020). Reprinted with permission from ref. 121. Copyright 2021 Elsevier.

In their three articles published following the progress according to the years 2016, 2019, and 2021 under the title of nanoparticles in the clinic, Anselmo and Mitragori listed the clinically approved nanoparticle therapies and diagnostics in the European Medicines Agency (EMA) and Food and Drug Administration (FDA) field, as well as the nanoparticles that are not approved but are currently undergoing clinical research [diagnostic agents or therapeutics (*e.g.*, elements, small molecules, biologics), synthetic materials (*e.g.*, polymers), and biological molecules (*e.g.*, antibodies, peptides, lipids)]. Although nanoparticle drug delivery systems can be applied *via* different administration routes, the most interest, both preclinically and clinically, has focused on their intravenous administration. In a 2016 review, researchers listed over 25 nanoparticle medicines (nanomedicines) approved by the FDA and EMA for intravenous administration and over 45 unapproved but clinically evaluated nanoparticle therapies and diagnostics.^119^ In the following three years, two intravenously administered nanoparticles were approved by the FDA and EMA, and one intratumoral nanoparticle received market approval (CE); 75 clinical trials of previously reported intravenous nanoparticles and 15 new intravenous nanoparticle technologies entered clinical trials.^120^ In the 2021 update, the current clinical landscape was reviewed with the addition of two nanoparticles that received emergency use approval in 2020, over 30 new trials that were previously mentioned but not approved, and 35 new nanoparticle technologies that recently entered clinical trials (regarding 55 new trials). Accordingly, the nanoparticles and particle types that received official approval are shown in the Fig. 3.^121^ Lipid-based and inorganic nanoparticles constitute the majority of clinically approved nanoparticles. In addition to these systems, nine polymeric nanoparticles were approved and cited in the EU Innovation Network (EU-IN) Horizon Scanning Report.^122^ When the nanoparticles approved in terms of indication are evaluated, the first three are cancer, anemia, and imaging.

**Fig. 4:**
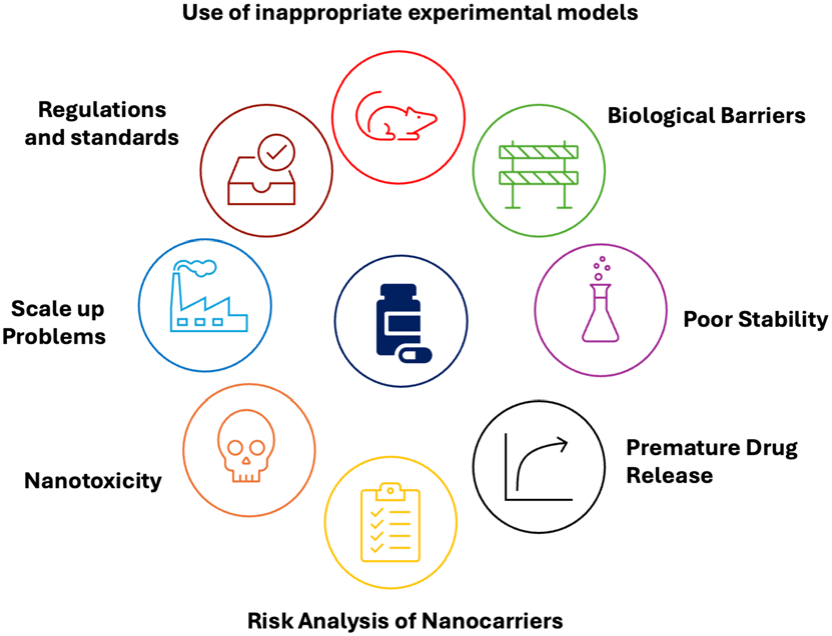
Challenges encountered in the transition of nano drug carriers from research level to patient use.

Namiot *et al.* (2023) stated that the number of clinical trials conducted since 2002 is 486, and when evaluated in terms of drugs, many clinical trials focus on the same active substances.^123^ The most investigated molecules are paclitaxel (23%), metals (11%), doxorubicin (9%), bupivacaine (8%) and various vaccines (8%). The number of nanoparticle drug products that have received official approval is quite low compared to the number of clinical trials conducted. This statistic is attributed to the presence of some problems that should be overcome in the clinical translation of nanoparticles (Fig. 4). This section will define these challenges, which can be characterized as large-scale problems affecting nano systems.

### 5.1. Premature release of active substance from nanoparticle drug delivery system

Nanoparticle drug delivery systems are designed to reduce side effects and precisely deliver drugs to their target organs and tissues. Nanoparticular systems are directly delivered to the systemic circulation and primarily taken up by the reticuloendothelial system (RES) *via* passive targeting. Although this is an advantage for applications targeting this system, access to other organs and tissues is not provided. Although active targeting is advantageous in this context, premature drug release (PDR) in the bloodstream before nanoparticle systems reach the target site emerges as a problem that needs to be overcome. PDR should be evaluated especially for anticancer drugs. Providing long-term circulation in the systemic circulation also increases PDR. Slow drug release can be an effective way to reduce premature release but also reduces the concentration of free drug in target cells. Ideally, a nanoparticle drug delivery system should provide an initial burst intracellular drug release without PDR. In this respect, nanoparticle systems designed for the stimulated drug release with endogenous (temperature, enzyme, reactive oxygen species, pH, and redox potential) and exogenous activators (temperature, light, X-rays, magnetic fields, and ultrasound) have significant potential.^124^ Li *et al.*^125^ prepared salinomycin-entrapped solid lipid nanoparticles surface modified with clathrin (CMSLN-SAL) to reduce PDR and ensure intracellular burst drug release. It was observed that as a result of a cage-like structure formation on the SLNs due to the triskelia structural feature of clathrin, the mechanical strength of the nanoparticles was increased, showing the capability of CMSLN-SAL against external pressure in the blood flow and internal pressure within the nanoparticles. When the drug release was tested in non-agitated plasma, ultrasound-agitated plasma, and 0.22 μm membrane filter under different flow rates for examining the effect of hydration-induced internal pressure, external pressure by blood agitation, and squeezing through vascular clefts on PDR, respectively, it was seen that PDR from CMSLN-SAL was approximately 60% less than that of non-clathrin-coated nanoparticles. In the cytosol medium containing HSC70, drug release was roughly the same in clathrin-coated and uncoated SLNs, indicating that the clathrin coat of internalized CMSLN particles was depolymerized by HSC70, providing burst release in cells. In intracellular uptake studies using HepG2 cells, the uptake of CMSLNs was also higher than that of SLNs by clathrin-mediated endocytosis.

Another approach to prevent PDR and burst release is drug–polymer conjugates and core- or corona-crosslinked micelles, where the drug is covalently linked to the carrier. The crosslinks or covalent bonds are designed to be sensitive to an intra- or extracellular stimulus. Modifications to the preparation methods of nanoparticle systems should also be evaluated for their effects on PDR. In addition, the thermodynamic stability of the carrier system also affects PDR.^126^ The low critical micelle concentration (CMC) values of the micelle forming copolymers provide thermodynamic stability of the systems. Due to this feature, the polymeric micelles can maintain their integrity even if they are highly diluted *in vivo*.^127^ However, premature degradation or the breakdown of the surface coating around the nanoparticles are other obstacles to the drug reaching the target tissue. The degradation of nanoformulations in systemic circulation and within the cell is an important parameter in evaluating their potential use in therapy. Therefore, accurately tracing their behavior and breakdown within the body is essential for successful drug delivery. Observation of *in vivo* fate involves techniques such as complex labeling procedures, fluorescence imaging and quantitative nuclear imaging (Positron Emission Tomography and Single Photon Emission Computed Tomography).^128^

### 5.2. Biological barriers

Upon administration of nanoparticles, overcoming the biological barriers in the human body is one of the major challenges that determines the clinical usefulness of these systems. The particle size and hydrophobicity of nanoparticle systems allow passive targeting to the RES system, while pegylation allows long-term circulation of nanoparticle systems in the blood without being taken up by the RES, and ligand conjugation allows active targeting to specific organs and tissues. Targeting efficacy varies depending on the ligand density bound to the nanoparticles and the conjugation strategy used. Studies have conflicting findings regarding how ligand density affects the biological behavior of a nanoparticle. This should be evaluated individually for each carrier.^129^ The positive surface charge of nanoparticles provides higher cellular uptake, while the negative surface charge minimizes nonspecific uptake from the negatively charged cell membrane and extends their half-life in the blood.^130^ The interaction between nanoparticles and biological barriers is also an important factor. The molecular interactions of nanoparticles, including cellular uptake and increased retention and permeability effects in tumor tissue, are a topic of focus. The effects of transport efficiency and targeting on the efficacy and safety of nanoparticles as nanomedicines, as well as their interactions with biological barriers, need to be well characterized. In this respect, imaging methods and quantitative measurements need to be developed to visualize and quantify the biodistribution of nanoparticles.^131,132^ Biological processes driving diseases operate at the nanoscale, linked to issues such as mutated genes, misfolded proteins, or viral and bacterial infections. By better understanding these molecular processes, we can rationally design engineered nanomaterials that specifically target the required site of action within the body.^133^

There are physiological and pathological differences between humans and animals, and it is uncertain how these differences will affect the *in vivo* behavior and efficacy of nanotherapeutics. The methods used in animal studies and human clinical studies also differ, and correlations could not be established between studies. For example, organ extraction and tissue removal for biodistribution cannot be applied in clinical studies.^131^ These reasons create a translation gap between animal and human studies. Nanoparticles also interact with biomolecules in the bloodstream and suspended cells in the blood. The nanomaterial composition and the suspension medium affect the extent of the interaction. Hydrophobicity, particle size and distribution, shape, surface area, charge, roughness, porosity, and presence of ligands or other moieties are several material properties that affect the relevant surface properties.^133^ Nonspecific adhesion of serum proteins and lipids causes the nanoparticles to be coated with a corona on their surface,^134^ which changes their biological effects and affects their cellular uptake, biodistribution, clearance, toxicity and immune response. Therefore, it is necessary to focus on protein coronas formed around nanoparticles.

Another important obstacle to the success of nanotherapeutics is the heterogeneity of biological barriers – systemic, microenvironmental, and cellular – across patient populations and diseases. Overcoming patient heterogeneity can be achieved through precision therapeutics in the form of personalized interventions with improved therapeutic efficacy. However, nanoparticle development continues to focus on the optimization of delivery systems with a one-size-fits-all-solution approach. Designing smart nanoparticle delivery systems that can overcome heterogeneous barriers is necessary to increase clinical success.^135^ The cited reference here includes a detailed review about biological barriers and is suggested for readers who wish to obtain more detailed information.

### 5.3. Nanotoxicity

**Fig. 5:**
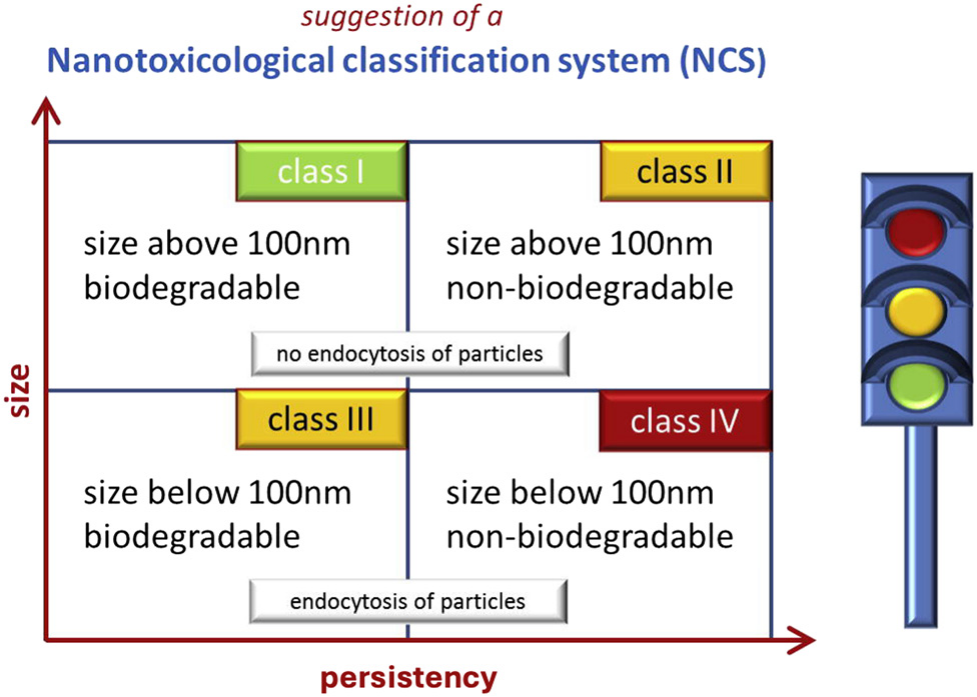
Nanotoxicological classification system (NCS) placing the particles according to size and persistency into four classes of increasing risk: I – no or low, II and III – medium, and IV – high risk. Reprinted with permission from ref. 137. Copyright 2021 Elsevier.

One of the issues that complicates the bench-to-market translation of nanoparticle systems is the nanotoxicology assessment. As mentioned above, the physicochemical properties of nanoparticles, such as their nanosize, shape, surface properties, and their composition, create unique differences in them and determine their toxicity profile. Depending on these properties, nanoparticles can cross cell membranes, the placenta, the blood–brain barrier, and other barriers in biological systems, resulting in unwanted accumulation and toxicity at the cellular level. Scientific studies on nanoparticles' toxicity at the molecular and cellular level show that oxidative stress, inflammation, genotoxicity and neurotoxicity play an intertwined role. Therefore, the safety and risk parameters of nanoparticles need to be defined very well.^136^ Keck and Müller (2013) proposed a simple but clear classification system based on particle size and biodegradability as the two most important parameters that determine the interaction of the nanomaterial with cells and its intracellular fate and effects (Fig. 5).^137^ The reason why 100 nm is taken as the limit for particle size is that they can be endocytosed and easily spread in the body. Biodegradable systems, on the other hand, have a low toxicity risk because they can be removed from the body. Nanoemulsions, liposomes, drug nanocrystals and lipid nanoparticles can be given as examples for class I, which includes particles with sizes above 100 nm and below 1000 nm and with no or lowest risk. However, these nanoparticulate systems can also cause unwanted side effects related to the immune system. The examples given in class I can also be given as sample nanoparticulate systems for class III if their sizes are below 100 nm. Nanoparticles in class II are non-biodegradable particles with sizes above 100 nm. Particles in class II constitute the medium risk group due to their persistence in the body and nanoparticles in class III constitute the medium risk group due to their possibility of reaching every cell in the body. Class IV includes non-biodegradable nanoparticles smaller than 100 nm (such as titanium dioxide and gold nanoparticles) that carry the highest risk of toxicity. The route of administration of nanoparticles can also affect potential nanotoxicity, as it determines their bioavailability in the body. The authors also noted that for the successful application of this classification system, nanoparticle systems must first be well characterized by standard methods and tools and evaluated in terms of bioavailability, pharmacokinetic pathways, persistency, degradation by-products, cell uptake, intercellular fate, and cell interaction.^137^

However, the characterization of nanoparticles remains poor due to the lack of standard and harmonized methods, the lack of a reference for comparison, and the fact that the doses used in experiments are sometimes not applicable to the biological system. Characterization of the *in vitro* and especially *in vivo* interactions of nanoparticles with biological systems is difficult. Cell viability, cytotoxicity tests for identifying oxidative stress; cell stress assays for gene expression are among the performed *in vitro* experiments. However, the main disadvantage of these tests is the difficulty in imitating interactions that may occur in the human body. In addition, *in vitro* measurements may not predict the pathophysiological response of the human body to exposure to nanomaterials. *In vivo* experiments conducted in animals may be impractical and unethical; in addition, the data obtained may not be able to establish a correct correlation between humans and animals. Toxicogenomics and quantitative structure–activity relationship models (QSAR) are methods that have been increasingly applied in nanotoxicology.^133,138,139^

### 5.4. Low stability

The stability of drug carrier systems is one of the most important issues in the formulation development step. During storage various physical instability problems such as fusion, aggregation, sedimentation, swelling and drug leakage are observed in the dispersion form of nanoparticles. Analysis of particle size, zeta potential and drug content, which are the basic characterization features of nanoparticles, allow the evaluation of the formulation stability. Addition of appropriate charge inducers and surface charge optimization can provide a solution to the aggregation and fusion problems. Another risk is the chemical degradation of nanoparticle components such as oxidation. Storing formulations as lyophilized in dry powder form is more advantageous in this respect.^92^

### 5.5. Scale-up and cost

Whether it is a conventional or innovative drug delivery system, scale-up in pharmaceutical production from Research&Development to large-scale manufacturing requires serious knowledge in areas such as formulation, pharmaceutical materials, biopharmaceutics, reaction kinetics, physical chemistry of macromolecules, homogeneous systems, manufacturing pharmacy, pharmaceutical engineering, evaluation of pharmaceutical dosage forms, principles of quality assurance, and regulatory side of drug product manufacturing and distribution. The manufacturing of a product meeting the specifications determined in the target product quality profile in a repeatable manner at full manufacturing scale in series depends on the success of the scale-up steps. Preparation of nanoparticulate systems involves multi-step and complex processes that make it difficult to control batch-to-batch changes both at scale-up and in full production. Examples of these processes frequently applied in nanoparticle formulation processes are high-speed homogenization, sonication, milling, emulsification, cross-linking, evaporation of organic solvents, centrifugation, filtration, and lyophilization. During initial formulation development studies at the laboratory or small scale, it would be useful to evaluate which of the alternative production processes would be beneficial in terms of scale-up of the product.

Small amounts of nanoparticles are prepared in laboratory, preclinical and clinical studies. Optimization is easy in these small-scale productions. However, in the transition to large-scale production, the manufacturing process parameters must be carefully adjusted to ensure the target product quality profile in a reproducible manner. The quality, safety, and efficacy of drugs containing nanomaterials can be very sensitive to processing conditions and production scale. Small changes in the manufacturing process may result in changes and loss of desired properties such as physicochemical properties of nanoparticles, biological properties and even therapeutic effects. Changes in the chemical structure and conformation of macromolecular, especially biological substances, may result in denaturation, cross-linking, coagulation and degradation.^132,140,141^ Therefore, defining the key process and formulation parameters is critical to achieve key properties and functions. Furthermore, nanoparticles are not simple mixtures but highly structured compounds. The structural integrity and physicochemical properties of nanoparticles must be preserved until they are converted into finished products.^124,142^ Nanoparticle drug delivery systems have many critical quality properties that determine their pharmacological effects, such as particle size and distribution, particle shape, surface properties (zeta potential, hydrophobicity, functional groups), drug content, drug release and stability. In addition, there are critical material properties such as the structure of the carrier system, molecular weight and distribution, degradation mechanism and degradation time, stability, and physicochemical properties to achieve these properties. Accordingly, quality-by-design (QbD) and process analytical technologies (PAT) will be suitable approaches for the development of nanomedicine formulations, evaluation, and control of nanomedicines.^143^

When the nanoparticles are aimed to be produced as a new drug product with a completely new drug (active ingredient), their cost is very high. As is generally the case in studies, when a nanoparticle system containing an existing active ingredient is aimed to be developed, it typically results in a higher drug price to cover the cost. Therefore, the lower-priced conventional drug product on the market is preferred by consumers. Even official agencies that provide reimbursement base on the conventional dosage form due to the low price. For these reasons, a careful cost-effectiveness analysis should be carried out in the commercial production of nanomedicines, and it becomes difficult to offer them as a treatment option.

### 5.6. Regulations and standards

The high complexity of nanomedicines with their heterogeneous structures and the variety of nanoparticle-based products make the complete physicochemical characterization difficult. Besides, the formal evaluation of not only original products but also generic or follow-on (referred to as nanosimilars) forms is challenging. There is a general lack of standards in the study of nanomedicines as a unique category of therapeutic agents. Nanoparticle-based drugs may contain more than one component that may affect the pharmacological behavior of the active substance. Compared to conventional dosage forms consisting of inactive ingredients and active substance, nanomedicines naturally require complicated formal strategies and more complex processes.^142,144^

However, there are reflection papers and guidelines published by FDA and EMA to assist manufacturers, suppliers, importers, and other stakeholders on drugs containing nanotechnology applications and nanomaterials. FDA guidance states that two points should be considered when assessing whether FDA-regulated products contain nanotechnology applications.^145^ These points address particle size and size-dependent properties or phenomena. Nanoscale materials (*i.e.*, those with at least one dimension in the range of approximately 1 nm to 100 nm) may exhibit different chemical, physical, or biological effects when compared to their larger counterparts. These effects may result from chemical, biological, or magnetic properties, altered optical or electrical activity, increased structural integrity, or other unique characteristics that are not normally expected or observed in larger materials of the same chemical composition. Such changes may raise questions about the efficacy, safety, performance, quality, and human health effects of nanotechnology products. The routes by which these products are administered are also critical to their evaluation. The second point focuses on the evaluation of size-dependent properties and phenomena in the case of sizes outside the range of 1 nm to 100 nm up to 1000 nm.^145^ According to the European Commission Recommendation, a nanomaterial is defined by the relative proportion of its constituent particles falling within a specific nanoscale range, based on their external dimensions in a particle number-based distribution. Additionally, materials with a volume-specific surface area less than 6 m^2^ cm^−3^ cannot be classified as a nanomaterial.^140^

FDA published detailed guidance in 2022 covering the manufacture and evaluation of human drugs (*i.e.* finished dosage forms), including biological products, in which a nanomaterial is included in the finished dosage form. This document discusses quality, nonclinical, and clinical studies as they relate to drug products containing nanomaterials (materials falling within points 1 or 2 described above) during product development and manufacturing. The guidance also includes recommendations for sponsors and applicants of investigational, premarket, and postmarket applications for products containing nanomaterials regarding the characterization, control, testing, and qualification of their nanomaterial components.^146^

The EU-IN Horizon Scanning Report titled “Nanotechnology-based medicinal products for human use” was published by EMA in 2025.^122^ This report stated that nanomedicines are regulated within the existing official framework for medicinal products and medical devices and that there is no separate EU framework for nanomedicines. The report emphasized the need to break the vicious circle of (1) regulators providing limited experience in regulating innovative nanomedicines, (2) only providing initial guidance, but (3) requiring product developers to submit an unspecified dataset, (4) heterogeneous/insufficient datasets available for safety and quality assessment of nanomedicines, then (5) the lack of robust datasets for regulators, which results in (6) delays in harmonization of regulatory practices and an increase in regulatory rejections. The EU-IN aims to promote/develop cooperation among stakeholders and reduce gaps. The steps to be taken and the paths to be followed are defined for this purpose.

The complexity of characterization of nanomedicines also makes it extremely difficult to prove the pharmaceutical equivalence and bioequivalence of follow-on products to reference nanomedicines. The currently defined generic paradigm is not applicable to follow-on nanomedicines. Classical bioequivalence testing (*i.e.*, measurement of plasma drug concentrations) may not represent the performance of nanomedicines due to different cellular uptake mechanisms in target tissues and route of administration; therefore, additional clinical studies may be required.^147^ When it is possible to demonstrate the pharmacological effect of nanomedicines at the intended target sites, disease models has been stated to preferably be applied.^142^ EMA has published guidelines for follow-on nanomedicines on a product basis (nano-sized colloidal iron-based preparations, intravenous liposomal products, nanoparticle iron medicinal product). Apart from these, regulatory approval processes need to be better defined and standardized across agencies to support adequate assessment of critical quality attributes as evidence of therapeutic equivalence of follow-on nanomedicines to reference nanomedicines for patient safety and efficacy.^147^ Advanced international collaboration is ongoing to ensuring the safe development of nanomedicines used for drug delivery. For instance, GoNanoBioMat project primarily aims to use the “safe-by-design” (SbD) approach, provide guidelines for it and address the regulatory landscape and product lifecycle safety.^148^

## 6. Conclusions

NRF2 modulators hold promise as therapeutic agents for various diseases but overcoming formulation challenges and conducting thorough research are essential for their successful development as effective and safe drugs. In order to prevent the low bioavailability, poor solubility and undesirable toxicities of NRF2 modulators, the use of nanoparticles has great potential in terms of transition to the clinic. Literature data support the idea that nanomedicine-NRF2 modulator synergy may provide significant clinical effects, particularly in cancer and neurological diseases. However, the transition of nanodrug carriers to clinical use also carries many risks, including the lack of regulation, difficulties in standardizing large-batch manufacturing, potential nanotoxicity *etc.* Despite these challenges, the progress of ongoing studies clearly indicates the feasibility of this synergy. Recent advances in nanocarrier engineering and bioresponsive nanomaterials,^149^ along with emerging technologies such as biomimetic systems (exosome-derived nanoparticles) are also creating a promising and open subfield for future investigation on the administration of NRF2 modulators.

The clinical translation of nanoparticle-based drug delivery systems could be significantly accelerated by establishing robust standards and clear regulatory pathways. A crucial first step involves the rigorous application of Good Manufacturing Practice to standardize production, ensuring consistent characteristics across different batches. Simultaneously, regulatory bodies such as the FDA and EMA must develop comprehensive guidelines specific to nanomedicine, addressing the unique challenges these materials present. It is essential to develop more comprehensive and long-term toxicity and safety studies, moving beyond traditional assessments to evaluate potential off-target effects and long-term accumulation.

## Conflicts of interest

There are no conflicts to declare.

## Data Availability

No new data were generated or analyzed in this review article. All data necessary to support this review are available within the published literature cited herein.
